# Candyfluidics: the art of fabricating micro- and nano-fluidic geometries using surface-deposited sugar scaffolds

**DOI:** 10.1039/d5lc00710k

**Published:** 2025-09-16

**Authors:** Tochukwu Dubem Anyaduba, Jesus Rodriguez-Manzano

**Affiliations:** a Department of Infectious Disease, Imperial College London London UK t.anyaduba@imperial.ac.uk j.rodriguez-manzano@imperial.ac.uk; b Department of Biotechnology, Hezekiah University Umudi Imo State Nigeria

## Abstract

We present Candyfluidics, a rapid and low-cost method for fabricating micro- and nanofluidic devices using sugar mixtures patterned by screen-printing. The process (from screen preparation to PDMS casting) takes less than 30 minutes, supports parallel production of multiple chips, and exploits household materials and simple technology widely available in low-resource regions. As a proof of concept, we fabricated flow-focusing chips and validated them by generating pressure-driven water-in-oil droplets with volumes from 0.2 to 1.22 nL. We further demonstrated the utility of the fabricated chips by performing digital droplet loop-mediated isothermal amplification to detect dengue virus type 1 nucleic acids at femtomolar concentrations (85 copies per μL). By lowering the cost and technical barriers to device prototyping, Candyfluidics offers an accessible approach to microfluidic manufacturing with potential for point-of-need applications for global health interventions.

## Abstract 2

From sugar to chips: reducing the barrier to microfluidics advancement in LMICs.

## Graphical abstract

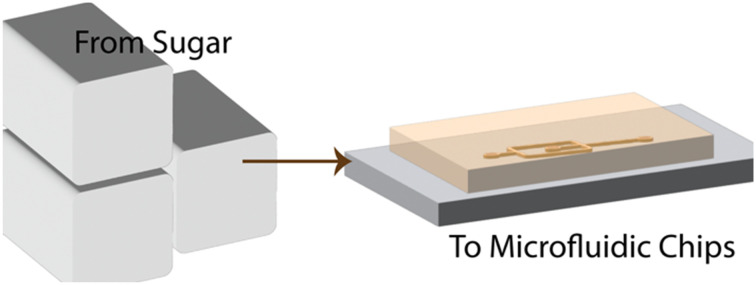

## 1. Introduction

**Fig. 1:**
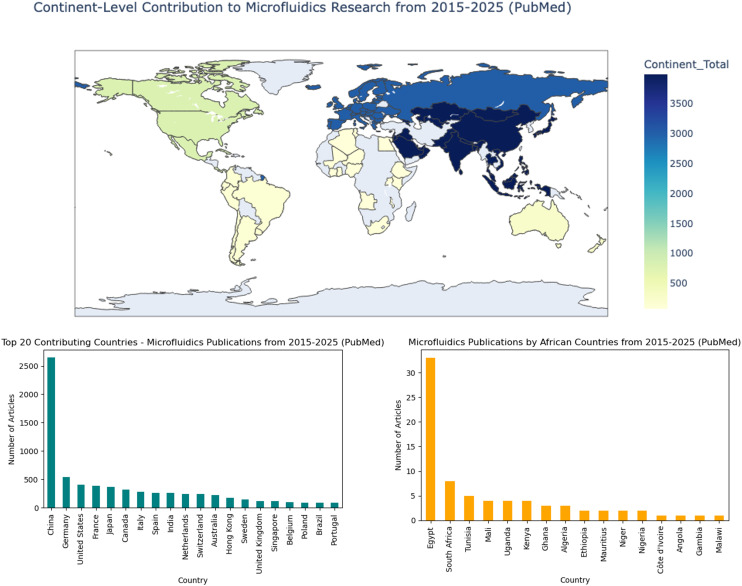
Growth of microfluidic research from 2015–2025, showing contributions (publications) by continent and country. This figure was generated from 8177 research articles indexed in PubMed under the keyword “microfluidic”. Out of the generated results, only 73 are linked to an author or co-author with an affiliation to African institutions, and just 32 are linked to sub-Saharan institutions.

Global health initiatives aimed at eradicating life-threatening diseases are futile without technological security and equity. For developing regions of the world such as sub-Saharan Africa (SSA), poor adoption, and very limited life-span of advanced medical instrumentation informs the need for alternative approaches. As embodied in WHO's (RE)ASSURED criteria,^1,2^ such alternatives should be fit for the region's context, hence the recommendation of miniaturization through microfluidic technologies.^3,4^ An evaluation of democratized research leading to the development of microfluidic platforms over a decade spanning 2015 to 2025 suggests the presence of an economic barrier (Fig. 1).

An extensive comparative analysis of microfluidic device fabrication methods identified that 3D printing has the lowest entry barrier in terms of running and equipment costs.^5–7^ In most low- and middle-income (LMICs) regions, however, 3D printing may still be capital-intensive, with equipment costs ranging from approximately $300 for basic setups to over $20 000 for specialised instruments such as the ProFluidics285 by CADWorks3D. Moreover, their operation requires prolonged availability of electricity which is a major challenge in many LMICs. This is especially true for regions in SSA, where approximately ∼41% of the population (∼525 million people) live in extreme poverty.^8^ The availability of low-cost, low complexity fabrication methods could increase access, and the advancement of miniaturized biomedical devices in SSA and other lower-income regions.

Low-cost methods such as paper-based systems have been extensively explored.^9–11^ However, they are limited by issues such as inefficient flow control,^12^ and handling requirements for imbibition maintenance. More importantly, biomedical processes such as droplet generation, which require unrestricted fluid flow and better fluid manipulation efficiency, cannot benefit from paper-based technologies. Hence, the need for alternative technologies for fabricating open channel, microfluidics-based architectures. Unfortunately, other inexpensive protocols for fabricating microfluidic devices such as shrinky-dinks,^13,14^ and soft lithography using etched printed circuit boards (PCB)^15^ present limitations. These limitations are exemplified by the complexity of predetermining the 3 dimensional (*X*, *Y*, *Z*) shrinking factors of shrinky-dinks in response to baking temperature, exposure time and the need for procedural and equipment precision.^14^ The user must first pre-determine *X*–*Y* shrinkage and its concomitant effect on channel *Z*-height *via* a calibration step using the intended shrinky-dink film lot, and baking conditions. More so, during fabrication, microfluidic channels formed from shrinky-dinks are prone to channel occlusion due to wall collapse or from undebrided materials left during initial channel scoring. There is also very limited control of the height of fluidic channels formed from etched PCBs and shrinky-dinks. For etched PCBs, the channel height is limited to the thickness of the copper layer in commercially available PCB boards. This limits the user's ability to tune the depth profile of the formed microfluidic chips. On the other hand, the dimensions of shrinky-dink chips are inversely coupled such that during baking, the channels shrink in the *X*–*Y* direction but thicken in the *Z*-direction. The depth of the channels after shrinking depends on four factors: depth of the initial channel before baking, thickness of the starting material, *X*–*Y* shrink ratio, and density of the material after shrinking. Other methods such as Pysanky require extrusion of wax using a modified *XYZ* plotter to create microfluidic patterns.^16^ Pysanky is limited by the need for continuous heating, which requires power and technical complexity of multiaxes control. Microfluidic channels resulting from the wax prints also pose the risk of increasing the hydrophobicity of the channel walls thereby impacting fluid flow. Most importantly, the aforementioned alternatives involve the use of resources or materials that are not readily available in some of the regions in great need, particularly in SSA.

In this work, we present a low-cost method for fabricating microfluidic structures using a common household material, sugar. The approach, termed Candyfluidics, leverages the principles of candy sculpting by heating a mixture of sugar and corn syrup to hard-crack stage (∼150 °C). This process, commonly used in the film industry for producing breakable glass props, is adapted here for microfluidic fabrication. In contrast to traditional sugar glass sculpting, the heated candy mixture is deposited onto substrates to form intricate microfluidic channel architectures. These sugar-based microfluidic structures are then imprinted onto materials such as polydimethylsiloxane (PDMS) or photosensitive resin, thereby producing functional microfluidic chips.

## 2. Materials and methods

### 2.1. Candy deposition: screen printing

**Fig. 2:**
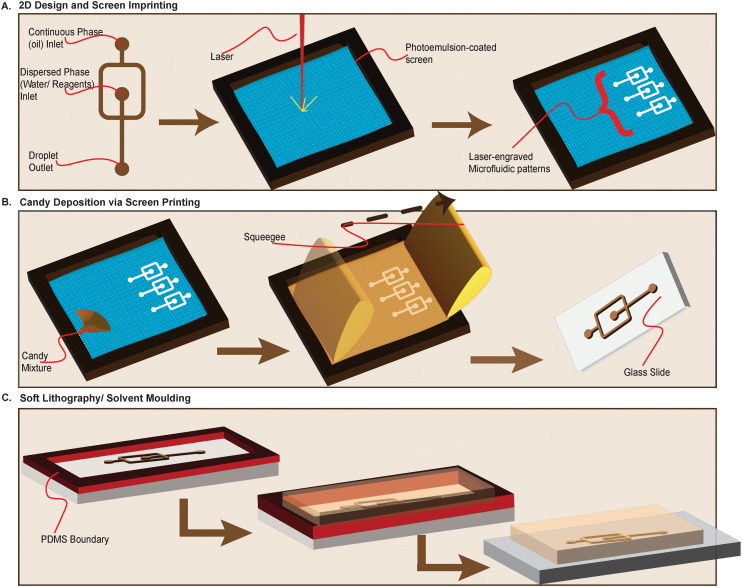
Candyfluidic chip fabrication process using screen-printing. A (Left to Right): Computer-aided design of a flow-focusing microfluidic architecture is drafted and etched on 100-mesh screen using a laser cutter. The frame of the mesh is aligned with the substrate (microscope slide) using a screen-printing kit from XTool. B (Left to Right): After a laser cutter is used to etch the intended design on the screen printer, the candy mixture is gently poured onto the mesh. A squeegee is used to force the candy mix through the laser-etched areas of the screen to imprint the design onto the slide. C (Right to Left): A bounding box (red) is used to define the dimensions of the chip (width, length, height) before the PDMS or photosensitive resin is poured. After pouring, the resin is allowed to harden before the cured PDMS is peeled to enable re-use of the candy print. Once peeled off, inlet and outlet holes can be installed using a biopsy or blunt needle. The imprinted rounded inlets shown on panel A guide the installation of 0.8 mm through holes on the PDMS.

Although any screen-printing technique can be used, in this proof-of-concept study we employed laser-aided screen-printing (XTool) to engrave 2D microfluidic designs onto a 100-count mesh screen. As shown in Fig. 2, a slurry of candy glass mix is printed onto a glass slide using the laser-engraved mesh and allowed to cure. After the candy has cured, PDMS (Dow) or photosensitive resin (Elegoo) is cast on top of the printed candy channel and allowed to set. Once cured, the PDMS can be either peeled to reveal the channel imprint or perforated to allow flushing of the candy from the channels. The printed candy channels can be stored at room temperature for up to 3 weeks before further use.

### 2.2. Candy deposition: spin-coating

**Fig. 3:**
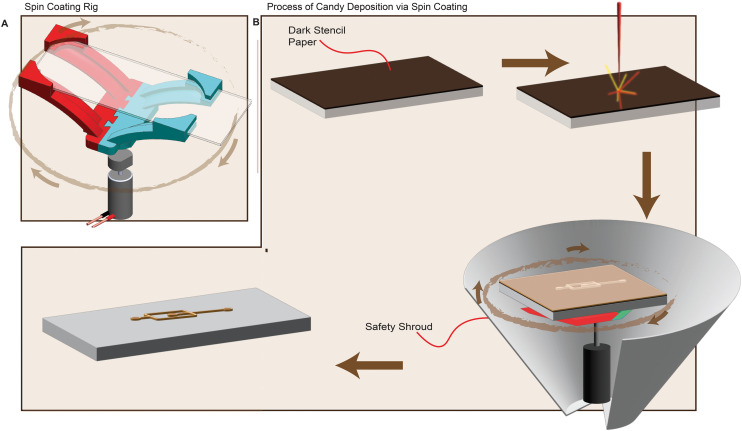
The process of depositing candy mixture onto glass slides *via* spin-coating A: exploded CAD of the spin-coating rig showing the eccentric rotational motor (ERM), slide holder, and slide. B: An opaque material (masking paper) is used to cover the slide to allow laser etching of the intended design. A laser is used to score the intended design. The scored design is peeled to expose the slide surface. The slide is attached to the shaft of a DC motor to enable even spreading of the candy mix. After the spin-coating is completed, the masking paper is peeled, revealing the deposited candy. Note that the soft lithographic process is omitted as it has been shown in Fig. 2C.

As an alternative to screen printing, we explored the use of spin-coating to deposit the candy mix. The spin-coating rig was designed using SolidWorks CAD software and constructed in-house. As shown in Fig. 3A, a 5 V DC eccentric rotational motor (ERM) was connected to a 3D-printed slide holder. Prior to spin-coating, an opaque adhesive tape (mask) was used to cover the microscope slide. This enabled the use of a laser to score or etch the microfluidic geometry onto the slide. Spin-coating enabled control of the channel height, which is determined by the thickness of the mask material. As a safety measure, a safety shroud is used to cover the spin-coating rig during each run.

### 2.3. Chip fabrication

#### 2.3.1. Candy mix

The candy mix was formulated using a 10 : 1 weight-ratio combination of sugar and corn syrup reconstituted in water. The resulting mixture was heated to 150 °C and stirred to enable complete dissolution of the sugar and evaporation to the desired consistency. The mixture for screen printing was evaporated to obtain a high-rheology consistency (similar to condensed milk), which prevented bleed-through the engraving mesh. The spin-coating mixture had a slightly lower rheology (similar to honey) to allow rapid distribution on the slide.

#### 2.3.2. Soft lithography

After printing the candy mix on a glass slide, it is allowed to dry (harden). Then, a 2.5 mm thick 3D printed boundary wall (50 mm × 21 mm × 4 mm – *L* × *W* × *H*) is taped around the print for PDMS casting. The PDMS was reconstituted at a resin to curing agent ratio of ∼7 : 1. Although a similar ratio has been shown to impact the tensile properties of PDMS,^17^ it was used here to promote fast curing and to ensure that the PDMS does not remain tacky, facilitating easier handling. Curing the PDMS at high temperatures^17^ caused the candy to melt and resulted in loss of channel definition. Therefore, the chips were cured at 50 °C for 1 hour. Curing for about 5 hours at 50 °C resulted in a formed channel on PDMS, the candy melted and could not be reused for further PDMS casting. During curing, we ensured that the slide was not in direct contact with the heated surfaces of the incubator to prevent rapid softening of the candy patterns. Once cured, the PDMS was peeled off, and fluid inlet and outlet holes were created using a biopsy punch according to the printed design. The chip fabrication was completed by bonding the moulded PDMS to glass slides after surface flame activation.^18^

#### 2.3.3. Solvent moulding

Although PDMS-based chips can be reused, they are difficult to clean since they are irreversibly bound to the chip substrate and are hydrophobic. We created glass-like chips through photopolymerization and solvent moulding using Elegoo stereolithography (SLA) 3D printer resin. These enabled easy cleaning and reuse of chips once the bottom seal (adhesive) is peeled off. Additionally, we also explored the feasibility of imprinting the printed candy on polycarbonate using polycarbonate plastic slurry. Details of how the polycarbonate slurry was made are available from the authors upon request.

#### 2.3.4. Dimensional analyses and conformance evaluation

To assess the dimensional conformance of the candy prints to the nominal design parameters, stereo micro-graphs of the prints were captured using a Keyence VHX-7000 digital microscope. The dimensions (length and width) of the prints were determined using FIJI (ImageJ).

### 2.4. Droplet microfluidics

#### 2.4.1. Droplet generation

We demonstrated the feasibility of generating water-in-oil droplets using flow-focusing chips developed with Candyfluidics. The continuous phase (CP) consisted of light mineral oil (Sigma-Aldrich) with 5% v/v EBIL EM 90 (Evonik) as the surfactant. The dispersed phase (DP) consisted of phosphate-buffered saline with 0.05% v/v Tween 20 as the surfactant. The DP was stained with fluorescein isothiocyanate (FITC) dye to aid micrography and image analysis. The fluids were pressure-driven using disc pumps (The Lee Company). The pumps were set up with PID-controlled settings (*P* = 5, *I* = 10, *D* = 0) and manual setpoints of *P*_cp_ : *P*_dp_ equal to 390 : 150, 320 : 150, and 320 : 160. These setpoints corresponded to driving pressure ratios (*P*_dp_/*P*_cp_) of 0.39, 0.47, and 0.50, respectively.

#### 2.4.2. Droplet digital loop-mediated isothermal amplification (ddLAMP)

The feasibility of applying Candyfluidics in single molecule detection for molecular diagnostics was further validated. Prior to ddLAMP, the limit of detection experiments using dengue virus serotype 1 (DENV-1) synthetic nucleic acid (G-block, IDT), containing a segment (308 bp) of the polyprotein gene, were performed using conventional real-time LAMP. The master mix consisted of 1× IsoAmp buffer, 6 mM MgSO_4_, 500 U mL^−1^ BST 2.0 WS, 1.4 mM dNTPs, and 0.2× evergreen dye. For the amplification reactions involving all six primer species, the primer cocktail consisted of 1.6 μM each of forward and backward inner primers (FIP and BIP), 0.2 μM each of forward and reverse outer primers (F3 and B3), and 0.4 μM each of forward and reverse loop primers (LF and LB), all purchased from Integrated DNA Technologies (IDT). Ten-fold dilutions of the synthetic nucleic acid ranging from 9.7 *×* 10^5^ to 97 DNA copies per reaction were amplified at 68 °C for 60 minutes using a Roche Lightcycler 96 thermocycler.

##### Droplet generation with DNA suspension

We passivated the flow channel using 1% BSA to prevent loss of DNA due to the hydrophobicity of the chip or tubing walls. We then generated nanolitre-scale droplets from the lowest concentration of DENV-1 synthetic nucleic acid, as determined by real-time LAMP. The same LAMP master mix recipe as described in bulk amplification was used. The droplets were incubated at 68 °C for 30 minutes and imaged using a confocal microscope (Leica SP5). The absolute concentration of DNA target in the dispersed phase was determined using Poisson statistics.^19^ Prior to Poisson calculation, we established a threshold to differentiate negative droplets from positive droplets. This threshold was determined as the maximum mean gray value of the droplets prior to amplification plus standard deviation. After amplification, a total of 7897 droplets were analyzed, resulting in 5447 negative droplets. The Poisson calculation considered a mean droplet volume of 4.3 nL formed under a driving pressure of 0.52 (*P*_cp_ : *P*_dp_).

The primer sets and synthetic nucleic acid sequence used in this work were designed in-house and are shown in Table 1.

LAMP primer and synthetic nucleic acid sequences used for detection of DENV-1

**Table:** 

Target organism/serotype: DENV-1	
Primer	Sequence (5′–3′)
DENV-1-F3	GATGGAGCAGAAAAATGC
DENV-1-B3	TGCGAACATTGGTCTCA
DENV-1-F2	ATGACTGGAACATTGGCTGTG
DENV-1-B2	TTCTGAAAGTGGCCATCAAAG
DENV-1-F1c	CCTGATCAGATCATTCCATGTCAAT
DENV-1-B1c	TCATGGTTGGAGCCAACG
DENV-1-LF	TGTCCCATTGTGAGAAGGAGGA
DENV-1-LB	CAGACAAGATGGGGATGGGAAC
DENV-1-FIP	CCTGATCAGATCATTCCATGTCAATATGACTGGAACATTGGCTGTG
DENV-1-BIP	TCATGGTTGGAGCCAACGTTCTGAAAGTGGC CATCAAAG

**Table:** 

Sequence (5′–3′)	
DENV-1-polyprotein (POLY) gene	TCAATAATGATCGAAGAGGTAATGAGATCCAGATGGAGCAGAAAAATGCTGATGACTGGAACATTGGCTGTGTTCCTCCTTCTCACAATGGGACAATTGACATGGAATGATCTGATCAGGCTATGTATCATGGTTGGAGCCAACGCTTCAGACAAGATGGGGATGGGAACAACGTACCTAGCTTTGATGGCCACTTTCAGAATGAGACCAATGTTCGCAGTCGGGCTACTGTTTCGCAGATTAACATCTAGAGAAGTTCTTC

## 3. Results

### 3.1. Dimensional conformance: screen printing

**Fig. 4:**
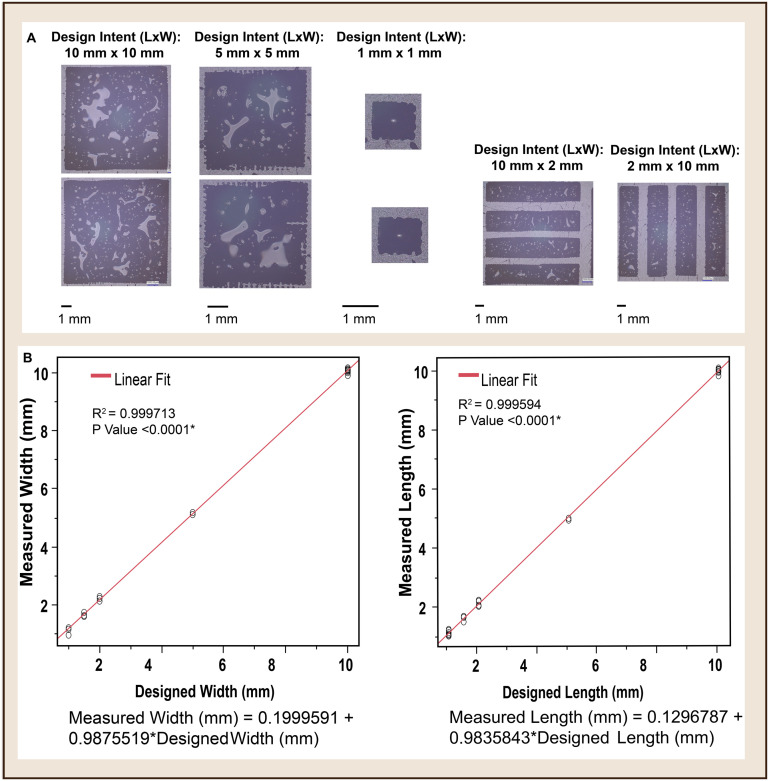
A: Dimensions of design iterations used to screen the process for dimensional conformance. (Right) Micrograph of the candy prints replicating the design specifications. B: *X*–*Y* plot assessing the conformance between measured length and width of the candy prints against the design intents. This shows a linear fit with *R*^2^ value ∼0.99 for both assessed parameters (width and length).

As shown in Fig. 4A, the edges of the candy prints were jagged due to the screen mesh definition and consistency of the candy mix. It can also be noticed that the surface of the prints was uneven due to bubbles. Both the jagged edges and bubbles were later improved by reducing the candy mix to paste-like consistency. Assessment of the dimensional conformance of the prints (Fig. 4B) showed strong fidelity (*R*^2^ value ∼0.9999) between the design intent and the printed candy with *P*-value *<* 0*.*0001. The tolerance within each analysed dimension was within ±0.06 mm.

**Fig. 5:**
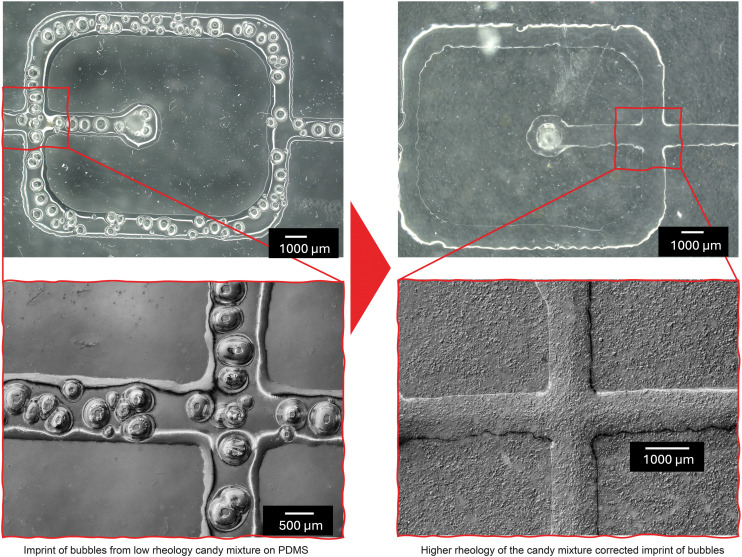
(Top) Flow focusing microfluidic chip fabricated from (Left) low rheology candy mix (Right) high rheology candy mix. (Bottom) 3-Dimensional depth composition of the fluidic channels from PDMS.

Further analysis characterizing the features of microfluidic channels fabricated from screen-printed candy was carried out. While the dimensional accuracy of the screen-printed candy was maintained, the surface of the channels were irregular due to imprint of bubbles from the prints (Fig. 5). The bubbles were found to be linked to the consistency of the candy mix (low rheology mix). A faster squeegee rate of the low rheology mixes also led to increased bubbles.

**Fig. 6:**
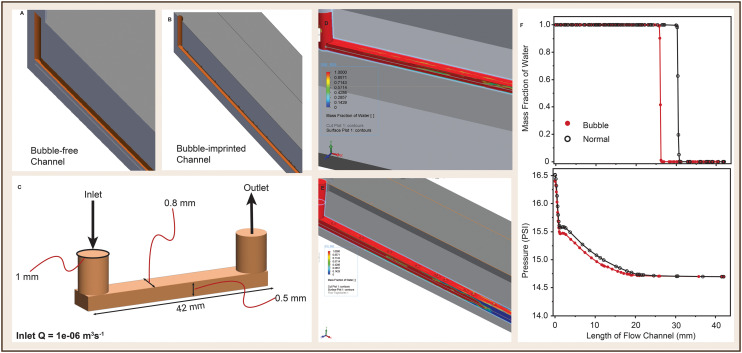
Computational evaluation of the effect of channel-imprinted bubbles on flow characteristics. (A and B) 3D CAD models for computational simulation to study the effect of bubbles on fluid flow. (C) Internal dimensions of the flow channels of A and B. (D and E) Surface plot of A and B showing mass fraction of water in the fluidic channels. F shows *XY* plots of mass fraction of water (top) and pressure (bottom) resulting from bubble-free channel (black) and bubble-imprinted channel (red).

Using SolidWorks Flow Simulation software, we simulated the effect of the bubble imprints on the PDMS to fluid flow in the microfluidic channels (Fig. 6). As expected, the *X*–*Y* plot of the fluid behaviour across the flow channel suggest a reduction in volumetric flow rate because of the increased channel volume due to the bubble imprints (Fig. 6F). This is evident from lower mass fraction of water in the fluid in the bubble-imprinted channel (red) in comparison to the normal channel (black). Increase in volume of the channels also resulted in the difference (reduction) in fluid pressure.

### 3.2. Droplet generation

**Fig. 7:**
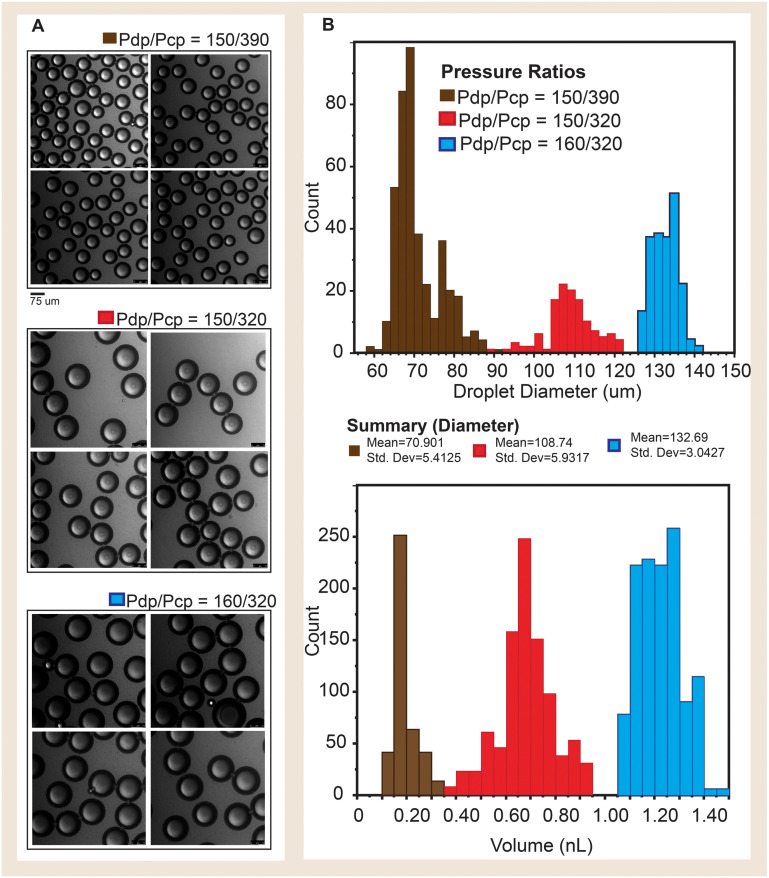
A. Confocal micrographs showing droplets generated at varying driving pressure ratios. B. Histograms showing the distribution of C-top droplet volume (nL) and C-bottom droplet diameter (μm).

The fabricated flow focusing chip was used to generate droplets at varying driving pressures corresponding to ∼0.39, 0.47 and 0.50. These yielded droplets with average diameters of 70.90 ± 5.41 μm (∼0.20 nL), 108.74 ± 5.93 μm (∼0.68 nL), and 132.69 ± 3.054 μm (∼1.22 nL) respectively. The polydispersity index of the generated droplets was calculated to be 8, 5, and 2% respectively for the fluid driving pressure of 0.39, 0.47 and 0.5 (Fig. 7).

The increase in droplet size corresponding to the increase in driving pressure corroborates findings from other researchers^20,21^ and introduces new evidence of the same for flow-focusing channels.

### 3.3. ddLAMP

**Fig. 8:**
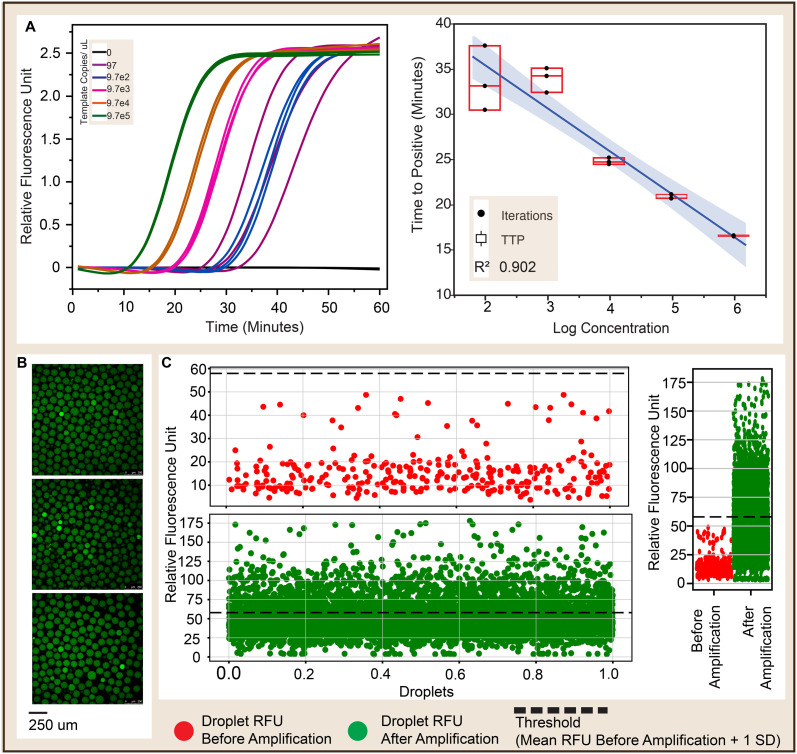
A. [Left] Amplification curve showing mean sigmoid (Logistic 4P) fits of different concentrations of Dengue virus 1 POLY gBlock. Each concentration was amplified in 3 replicates. Minimum concentration tested was ∼97 copies per μL in 10 μL bulk volume. [Right] Standard curve showing a linear relationship between concentration and time to positive *R*^2^ = 0*.*902 of the replicates B. Micrographs of ddLAMP droplets showing negative (green) and positive (brighter green) droplets. [C. Left–Top] Dot plot of gray value of droplets before amplification and [Left–Bottom] dot plot of droplets after amplification. A threshold line cutting off the negative droplets from the negative droplets is defined in the dot plots. Threshold is defined as the sum of the max value of the droplet gray values (before amplification) and the standard deviation of the the droplet gray values (before amplification). C. [Right] Combined dot plot of droplets before and after amplification.

Prior to evaluating the application of the chips in ddLAMP, using conventional real-time LAMP, we determined the minimum concentration of dengue virus POLY gBlock that could be amplified efficiently using our primers. As shown in Fig. 8A, increase in the target DNA dilution resulted in decreased efficiency in amplification. This is evident from the corresponding increase in the standard deviation of the time to positive of the replicates. The efficiency of amplifying very dilute target concentrations can be improved by decreasing the mean free path of the 6 LAMP primer species and the target molecule. This can be ensured *via* reduction of the reaction volume. Thus, following the significant decrease in efficiency at ∼97 copies per μL (191 femtomolar) (Fig. 8A (left)), ddLAMP was run at the same concentration to determine absolute count.

The confocal micrographs (exemplified in Fig. 8B) of the generated droplets were first segmented using Arivis cloud AI image segmentation (instance). The segmentation files were then used as mask to enable feature recognition and extraction using skimage Python libraries. From each micrograph, the droplets were characterized for diameter (0.657 pixels per μm), volume (nL) and mean gray value (fluorescence intensity).

The mean gray values of the droplets (before amplification, Fig. 8C [top]) were used to establish a threshold to differentiate between positive and negative droplets. Conventionally, template-free droplets were used to establish a cut-off between positive and negative droplet. This, however, does not replicate the standards established with bulk amplification. Our method introduces a more realistic differentiation between negative and positive droplets by establishing a threshold with template-carrying droplets before amplification.

Using the Poisson equation, we determined the absolute concentration of the dilution to be 0.3714 copies per droplet (85.51 copies per μL). Thus, validating the application of Candyfluidics in both droplet generation and detection of molecules below bulk LAMP LOD.

## 4. Discussion

### 4.1. Significance of this innovation

In this paper, we present the cheapest and most accessible method for fabricating microfluidic structures which explores household materials such as sugar. We showed the feasibility of generating nanolitre-scale droplets using Candyfluidic chips. While this technology presents significant ease due to the accessibility of constituting materials, and equipment, a few limitations remain present.

### 4.2. Limitations

#### 4.2.1. Candy deposition

For our proof-of-concept, 100 mesh screens were used to create the candy patterns. This resulted in imperfect channel boundaries. This could be improved by using higher resolution screens (for example 200 mesh). Also, laser-assisted screen printing was used for this demonstration. Thus, the geometric conformance of the prints to the design is influenced by the laser. According to XTool, the diode laser has a resolution limit of 0.0036 mm, 0.01 mm motion accuracy. Thus, the theoretical (expected) tolerance should be 0.0136 mm – the sum of the laser resolution limit and its motion accuracy, however, engraving will be inconsistent with features less than the wire spacing of 0.254 mm. Considering this, design features less than 0.254 mm or positioned between mesh gaps, may have much lower conformance. With traditional screen printing *via* UV exposure, the near-perfect conformance between design and prints may not be the same, especially with imperfect optical masking.

In addition to the possibility of using traditional screen printing, spin-coating of the candy mix over a precut pattern on the slide may also suffice. However, the temperature of the spin-coating chamber should be kept high enough to prevent fast cooling of the candy, which would result in uneven distribution of the candy on the substrate.

#### 4.2.2. Candy mix

The consistency of the candy mix is transient and temperature-dependent. It also hardens (cools faster) on contact with the screen printing mesh. This limits the throughput using the screens. If high-throughput runs are desired, a temperature-controlled environment may be ideal. It is also important to lift the screen away from the print when the candy is still fluidic; this helps ensure the edges of the prints stay intact. Care must also be taken to prevent scalding from exposure to the hot candy mix.

#### 4.2.3. Channel definition

A major advantage of Candyfluidics is the feasibility of fabricating fluidic channels with high aspect ratios (*W* : *H* dimensions) while retaining nano-volume handling characteristics. Thus, the need for micron-scale *X*–*Y* dimensions may not be apparent. However, while the *X*/*Y* dimensions can be predetermined by design, the height of the screenprints can mostly be pre-estimated following the pillar theory^22^ (eqn (1)).1
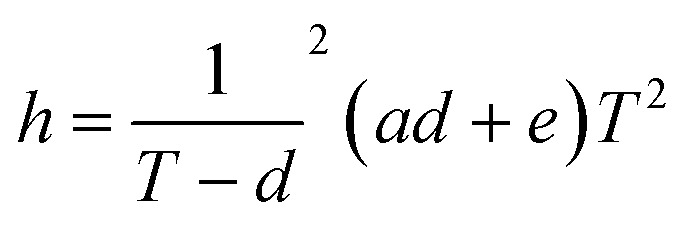
where *h* = channel height, *T* = mesh count (100), *d* = thread diameter, *a* = screen weaving tension, *e* = emulsion (candy) thickness.

A limitation to this, however, is that certain manufacturer-specific variables for XTool screens are unknown. Since XTool screens are not fully characterized, some manufacturer-dependent variables in eqn (1) such as “*d*” and “*a*” are unknown. More so, the candy mix is a thermo-thinning fluid, thus “*e*” is undetermined. These limitations make it challenging to predefine the channel height, “*h*” without additional specialized equipment. Other channel definitions as explained in section 4.1 can be improved using screens with higher resolution (high mesh count) and mesh gap closer to the laser resolution.

## 5. Conclusion

This study demonstrates the feasibility of developing nanofluidic architectures without the need for expensive machinery or advanced technical skills. Our results confirm that surface-deposited sugar scaffolds can enable the advancement of miniaturized systems for molecular diagnostics at femtomolar scale. Considering unbridled access to sugar, and screen-printing technologies in LMICs (especially in sub-Saharan Africa), adoption of Candyfluidics will bridge the resource gap. It will engender democratised advancement and adoption of microfluidics-based biomedical systems. While promising, Candyfluidics depends on a thermo-thining mixture of sugar and corn syrup which limits its throughput and large-scale adoption outside a temperature-controlled environment. Further research characterizing the optimal candy mix formulation and rheology, and predetermination of the candy-deposit height would significantly improve the robustness of Candyfluidics.

## Author contributions

T. D. A.: conceptualization, methodology, investigation, data curation, formal analysis, visualization, writing – original draft, writing – review and editing. J. R. M.: conceptualization, writing – review and editing, resources, funding acquisition.

## Conflicts of interest

There are no conflicts to declare.

## Data Availability

Data for this article, including micrographs and Python codes, are available at Zenodo (https://doi.org/10.5281/zenodo.15984955).
